# 25-hydroxyvitamin D levels are inversely related to metabolic syndrome risk profile in northern Chinese subjects without vitamin D supplementation

**DOI:** 10.1186/s13098-022-00793-1

**Published:** 2022-01-29

**Authors:** Hanyuan Xu, Guiyan Han, Linjie Wang, Huihua Ding, Chunyan Wang, Xiaochuan Ping, Caixia Dong, Dianxi Zhang, Yufei Dai, Naishi Li, Yufeng Li, Hongbo Yang, Huijuan Zhu, Hui Pan, Fengying Gong, Jichun Chen, Xiaoping Xing

**Affiliations:** 1 Key Laboratory of Endocrinology of National Health Commission, Department of Endocrinology, Peking Union Medical College Hospital, Chinese Academy of Medical Sciences and Peking Union Medical College, Beijing, China; 2 Department of Endocrinology, Beijing Friendship Hospital Pinggu Campus, Capital Medical University, Beijing, China; 3 Nutrition department, Fuwai Hospital, National Center for Cardiovascular Diseases, Chinese Academy of Medical Sciences and Peking Union Medical College, 167 Beilishi Road, Xicheng District, Beijing, China

**Keywords:** 25-hydroxyvitamin D [25(OH)D], Metabolic syndrome (MetS), Metabolic indices, Cross-sectional study, Longitudinal study

## Abstract

**Background:**

The comparatively low 25 hydroxyvitamin D [25(OH)D] levels have been reported in patients with metabolic syndrome (MetS). Herein we investigated the cross-sectional and longitudinal relationships between serum 25(OH)D levels and MetS risk profile in northern middle-aged Chinese subjects without vitamin D supplementation.

**Methods:**

A cohort of 211 participants including 151 MetS patients and 60 controls at 20–69 years of age were enrolled from suburban Beijing, China. The recruited MetS patients were subjected to diet and exercise counselling for 1-year. All subjects at baseline and MetS patients after intervention underwent clinical evaluations.

**Results:**

Serum 25(OH)D levels were significantly decreased in MetS patients. 25(OH)D levels were inversely related to MetS score, fasting blood glucose (FBG) and triglyceride-glucose index (TyG) after adjusting for cofounders (all *P* < 0.05). Participants in the lowest tertile of 25(OH)D levels had increased odds for MetS (*P* = 0.045), elevated FBG (*P* = 0.004) in all subjects, and one MetS score gain in MetS patients (*P* = 0.005). Longitudinally, the metabolic statuses as well as 25(OH)D levels of MetS patients were significantly improved (all *P* < 0.05), and the increase of 25(OH)D levels were inversely related to MetS scores, total cholesterol (TC), low-density lipoprotein cholesterol (LDL-C), FBG, and TyG, while positively related to high-density lipoprotein cholesterol (HDL-C) after adjusting for confounders.

**Conclusions:**

25(OH)D levels were significantly decreased in MetS patients, and it was negatively associated with metabolic dysfunctions at baseline and 1-year after. Metabolic aberrations of MetS patients were significantly ameliorated with 1-year follow-up counselling accompanying by notably elevated 25(OH)D levels.

## Introduction

Vitamin D is a crucial family of fat-soluble secosteroids which plays a vital role in regulating calcium and phosphate homeostasis in vivo*.* Other than maintaining bone health, vitamin D also possesses extra-skeletal effects, acting positively on cardiovascular system, pancreas and adipose tissue in an endocrine manner, and the elevated vitamin D levels help to prevent the occurrence and development of cardiovascular disease (CVD) [1], type 2 diabetes (T2DM) [2] and obesity [3]. However, vitamin D deficiency, characterized by serum 25 hydroxyvitamin D [25(OH)D] levels ≤ 20ng/mL, is highly prevalent worldwide. In China, it was shown that in adults dwelling in Gansu province (western China), an overall prevalence of vitamin D deficiency hitting 87.2% [4], while a study based on the child and young adolescent cohort in Beijing (northern China) concluded that 78.3% of the recruited samples were presented with vitamin D deficiency [5].

Metabolic syndrome (MetS) involves a cluster of metabolic dysfunctions including abdominal obesity, elevated fasting blood glucose (FBG), high blood pressure (BP), increased triglycerides (TG) and reduced high-density lipoprotein cholesterol (HDL-C). Studies have revealed that MetS was closely associated with various diseases [6], and subsequently contributes to all-cause mortality. It was reported that in mainland China, approximately 454 million Chinese adults were affected by MetS [7]. Due to highly overlapped risk factors (inadequate exercise, lack of sun exposure, etc.), increasingly abundant evidences associating vitamin D deficiency and MetS and its components were emerging over the years [5, 8, 9]. In vitro and in vivo studies also elucidated that vitamin D possesses the ability of regulating glucolipid metabolism. Biologically active metabolites of vitamin D_3_ ameliorated obesity by inhibiting the expression of lipogenic genes through inhibiting sterol regulatory element-binding protein (SREBP), which is a master transcription factor for lipogenesis [10]. Besides, activation of VDR prevented pathological dedifferentiation of pancreatic beta cells [11], and downregulation of VDR increased endothelial inflammatory response in preeclampsia [12], further shed light on the potential associations between vitamin D and MetS.

Individuals with MetS often present multiple metabolic perturbations, among which insulin resistance (IR) is a vital pathophysiologic mechanism of diabetes and may display decades prior to clinical diagnosis. Currently, the golden standard for measurement of IR is hyperinsulinemic euglycemic clamp, which is not of great convenience due to economical and ethical reasons. Therefore, in recent years, several novel surrogate indices including visceral adiposity index (VAI), lipid accumulation product (LAP), triglyceride-glucose index (TyG), triglyceride to high density lipoprotein cholesterol ratio (TG/HDL-C) and the metabolic score for IR (MetS-IR) have been introduced to identify IR in the absence of fasting insulin levels [13–16]. Notably, TyG have shown higher accuracy for IR compared with homeostasis model assessment of insulin resistance (HOMA-IR) [17]. As these indices were calculated based on combinations of waist circumference (WC) and indicators of blood lipid and glucose, it can be used as indicators for the condition of whole-body metabolism. There were a few studies conducted in different populations and regions have shown that they were also associated with MetS [18, 19]. However, the associations between these indices and vitamin D status are yet to be illustrated.

Vitamin D deficiency is highly prevalent and the relationship between vitamin D and MetS still controversial [20, 21]. Moreover, as MetS patients usually have lower vitamin D levels, and some studies suggest that vitamin D supplementation can improve the metabolic status of MetS patients [22]. It is also well established that lifestyle counseling such as diet and exercise interventions brings about metabolic benefits for MetS patients [23], but how vitamin D levels changes after diet and exercise interventions remains unknown, and whether the metabolic improvements are related to the changes of vitamin D status is not clear either. Therefore, in this study, we firstly cross-sectionally investigate the relationship between serum 25(OH)D levels and MetS risk profile in northern Chinese subjects, then explore the changes of metabolic traits and vitamin D levels and their relationships in MetS patients before and after 1-year diet and exercise interventions (1 year of intervention was selected in order to avoid the seasonal variation on vitamin D levels).

## Materials and methods

### Subjects

All study subjects were recruited from the suburban Beijing including MetS patients and controls. A total of 151 participants (aged 20-69 years) who met the MetS diagnosis according to the worldwide definition of the metabolic syndrome proposed by the International Diabetes Federation (IDF) in 2005 [24] : (1) central obesity: WC for men ≥ 90 cm or for women ≥ 80cm; and (2) plus at least two of the following items: (I) systolic BP (SBP) ≥ 130 mmHg and/or diastolic BP (DBP) ≥ 85 mmHg; (II) HDL-C < 1.03 mmol/L for males or < 1.29 mmol/L for females; (III) TG ≥ 1.70 mmol/L; and (IV) FBG ≥ 5.6 mmol/L and/or 2h-BG ≥ 7.8 mmol/L. Simultaneously, 60 control subjects (aged 20–64 years) who met the following inclusion criteria were enrolled in the same region. The inclusion criteria were as follow: (1) Annual income, nationality and lifestyle were matched with the patients in the MetS group; (2) BMI < 28 kg/m^2^; (3) Waist circumstance < 80 cm for females, < 90 cm for males; (4) Fasting blood glucose < 6.1 mmol/L; (5) Systolic blood pressure (SBP) < 135 mmHg, diastolic blood pressure (DBP) < 85 mmHg; and (6) Serum triglycerides < 1.7 mmol/L. All MetS patients were received diet and exercise counselling. Firstly, the frequency of follow-ups during the 12-month intervention were once every two weeks within the first 2 months, followed by once every month for another 4 months, then once every 3 months for the last 6 months. The interventions were including diet and exercise counseling. Secondly, the diet counseling was carried out by one trained dietician in accordance with the following protocol: (1) Calorie restriction: reduce the intake of high-calorie foods and the proportion of carbohydrate food, daily calorie intake is restricted to 1600 kcal for males and 1400 kcal for females; (2) Salt restriction: daily salt intake is restricted to 6 g per person (salt in seasoning is included); (3) Dietary calcium recommendation: daily intake of 250 mL milk, yogurt or 30 g of soybeans is strongly recommended; (4) Dietary fiber recommendation: daily intake of 400–500 g vegetable and 200 g fruit are recommended; (5) High cholesterol food restriction: daily intake of meat is restricted to 75 g and no more than 4 yolks a day are recommended; (6) Unsaturated fatty acid recommendation: replace the consumption of animal fat with plant oil as much as possible, deep see-dwelling fish is also recommended; and (7) Quitting smoking and alcohol are required. Thirdly, the exercise program was consisted of moderate intensity exercises. Each participant was equipped with a pedometer and required to walk 10,000 steps per day. In general, 20–30 min of outdoor fast walk after meal is recommended. The control or collection of data were conducted in accordance with standard protocol. For 5 subjects with elevated blood pressure (SBP ≥ 140 mmHg or DBP ≥ 90 mmHg) and blood glucose (FBG ≥ 7.0 mmol/L), 40–80 mg daily Telmisartan and 15 mg daily Pioglitazone were prescribed. These subjects were excluded in the analyses to avoid possible interference from medication. The study protocol was approved by the Clinical Medicine Ethics Committee of Fuwai Cardiovascular Diseases Hospital, Beijing, China (No. 110-3) for approval consistency. All participants provided informed consent before entering the study.

### Anthropometric measurements

Anthropometric measurements were performed by the same trained medical staff. Weight (Wt) were measured to the nearest 0.1 kg using an electronic scale, height (Ht) was measured to the nearest centimeter using a stadiometer in standing position and WC was measured as subjects stood with the tape measure held parallel to the ground at the midpoint of the line between the upper edge of the hip and the lower edge of the ribs, close to the skin without squeezing. BMI was calculated by dividing weight (kg) by height squared (m^2^). SBP and DBP were measured for three times utilizing a standard method and the mean value was taken.

### Biochemical measurements

Venous blood samples were taken from all participants after over-night fasting. Serum total cholesterol (TC), TG, HDL-C, low-density lipoprotein cholesterol (LDL-C), FBG, liver and kidney function were measured by routine automated laboratory methods in our clinical laboratory. 25(OH)D and parathyroid hormone (PTH) levels were determined by electrochemiluminescence method in our hospital.

### Calculations of metabolic indices

MetS score was calculated according to previous publication [25]. In brief, control subjects exhibit none of the 5 MetS components defined as MetS 0 score, and MetS patients with 3, 4 and 5 of the components defined as MetS score 3, 4, and 5, respectively. VAI, LAP, TyG [13], MetS-IR [16] and TG/HDL-C [26] were calculated using the following equations:

$$VAI(Men)=\left(\frac{WC(cm)}{39.68+(1.88\times BMI(kg/{m}^{2})}\right)\times \left(\frac{TG(mmol/L)}{1.03}\right)\times \left(\frac{1.31}{HDL-C(mmol/L)}\right)$$

$$VAI(Women)=\left(\frac{WC(cm)}{39.58+(1.89\times BMI(kg/{m}^{2})}\right)\times \left(\frac{TG(mmol/L)}{0.81}\right)\times \left(\frac{1.52}{HDL-C(mmol/L)}\right)$$

$$LAP\left(Men\right)=\left(WC(cm)-65\right)\times TG(mmol/L)$$

$$LAP\left(Women\right)=\left(WC(cm)-58\right)\times TG(mmol/L)$$

$$TyG=Ln\left(\frac{TG(mg/dL)\times FBG(mg/dL)}{2}\right)$$

$$MetS-IR=\frac{Ln[2\times FBG(mg/dL)+TG(mg/dL)]\times BMI(kg/{m}^{2})}{Ln[HDL-C(mg/dL)]}$$

$$TG/HDL-C=\frac{TG(mmol/L)}{HDL-C(mmol/L)}$$

### Statistical analyses

All analysis was performed using SPSS (version 25.0) for Windows. Data exhibiting skewed distributions were logarithmically transformed prior to analysis. Data were presented as mean ± standard deviation (SD) for continuous variables or median and inter-quartile range (IQR) for skew distributions, and counts (fractions) for categorical variables. Comparisons of anthropometric characteristics, clinical features and laboratory results between subjects with MetS patients and controls were performed using the Student’s t-test or general linear model with adjustment of age and gender. Associations between 25(OH)D levels and continuous metabolic parameters were evaluated by partial correlation analysis. 25(OH)D levels in subjects with different MetS scores were calculated and expressed as mean ± SD. Multiple logistic regression models were used for MetS or MetS components in relation to the tertiles of 25(OH)D, and odds ratios (ORs) and 95% CIs were calculated after adjusting for potential confounders. In longitudinal analysis, comparisons of anthropometric characteristics, clinical features and laboratory results of subjects between baseline and 1 year after were performed by paired T-test. Linear mixed effect model was built to assess the association between 25(OH)D levels (independent variable) and multiple metabolic parameters and indices (dependent variables). *P* value < 0.05 (two-sided) was considered as statistically significant.

## Results

### Comparison of clinical and biochemistry parameters between the controls and MetS patients

A total of 151 MetS patients (M/F: 71/80) and 60 controls (M/F: 20/40) were recruited in this study. As presented in Table 1, MetS patients had significant higher Wt, BMI, WC, SBP, DBP, TG, FBG, VAI, LAP, Uric acid (UA), Creatinine (Cr) and lower HDL-C levels when compared with the controls as expected (all *P* < 0.01). Metabolic indices including TyG, MetS-IR and TG/HDL-C were also significantly increased in MetS patients (all *P* < 0.001).

**Table 1:** Comparison of clinical characteristics of control participants and MetS patients

Variables	Controls	MetS patients	*P*
N (male/female)	60 (20/40)	151 (71/80)	0.070
Age (years)	44.02 ± 10.13	49.97 ± 7.73	** < 0.001**
Wt (kg)	59.45 ± 7.64	77.90 ± 11.86	** < 0.001**
BMI (kg/m^2^)	23.17 ± 2.38	29.25 ± 3.24	** < 0.001**
WC (cm)			
Male	79.75 ± 5.40	99.07 ± 7.28	** < 0.001**
Female	72.64 ± 5.41	89.25 ± 7.05	** < 0.001**
SBP (mmHg)	114.07 ± 10.58	141.83 ± 16.05	** < 0.001**
DBP (mmHg)	73.33 ± 7.75	90.61 ± 9.08	** < 0.001**
TC (mmol/L)	4.81 (4.25, 5.57)	4.91 (4.30, 5.73)	0.293
TG (mmol/L)	1.55 (0.93, 2.16)	1.80 (1.31, 2.45)	** < 0.001**
HDL-C (mmol/L)			
Male	1.09 (0.91, 1.32)	1.04 (0.89, 1.15)	** < 0.001**
Female	1.26 (1.09, 1.48)	1.18 (1.03, 1.29)	** < 0.001**
FBG (mmol/L)	5.06 ± 0.43	5.56 ± 0.55	** < 0.001**
UA (mmol/L)	224.48 ± 62.77	271.25 ± 83.00	** < 0.001**
Cr (mmol/L)	76.53 ± 8.90	81.13 ± 13.85	**0.005**
BUN (mmol/L)	4.69 ± 1.21	5.18 ± 1.44	0.051
VAI			
Male	2.25 (0.96, 3.07)	2.56 (1.97, 3.76)	** < 0.001**
Female	1.98 (1.06, 3.15)	2.64 (1.90, 3.73)	** < 0.001**
LAP			
Male	56.75 (24.09, 84.24)	67.33 (49.59, 90.90)	** < 0.001**
Female	38.09 (16.55, 59.30)	52.92 (38.16, 65.92)	** < 0.001**
TyG	8.14 ± 0.39	9.00 ± 0.52	** < 0.001**
MetS-IR	42.48 (36.01, 48.36)	45.59 (41.16, 50.23)	** < 0.001**
TG/HDL-C	1.26 (0.67, 2.04)	1.72 (1.12, 2.27)	** < 0.001**
25(OH)D (ng/mL)	16.63 ± 7.58	14.13 ± 3.65	**0.020**
PTH (mmol/L)	42.55 (34.25, 52.63)	43.1 (36.68. 52.63)	0.528

Data were expressed as ratio for categorical variables and mean ± SD for normally distributed continuous variables, median (IQR) for skewed distributed continuous variables. *P* values are from Student’s T-test or general linear model with adjustment of age and gender. Values in bold are of significance at *P* < 0.05

The mean levels of 25(OH)D in MetS patients were significantly lower than that of the controls (14.13 ± 3.65 *vs*. 16.63 ± 7.58 ng/mL, *P *= 0.020). The overall percentage of vitamin D deficiency (< 20 ng/mL) was 92.05% in MetS patients. However, PTH levels between two groups showed no statistical difference.

### The partial correlations between 25(OH)D and metabolic traits in all subjects

As displayed in Table 2 (Adjusted 1), 25(OH)D levels were significantly negatively correlated with MetS score, Wt, WC, DBP, TG and FBG, as well as TyG, LAP and PTH in all subjects after adjusting the age and sex. After further adjustment of BMI (Adjusted 2), 25(OH)D levels still remained significantly negatively associated with MetS score (*P* = 0.001), FBG (*P* = 0.003) and TyG (*P* = 0.034).

**Table 2:** Partial correlations between 25(OH)D and metabolic traits in all subjects

Variables	Adjusted 1		Adjusted 2	
	r	*P*	r	*P*
MetS score	− 0.250	** < 0.001**	− 0.227	**0.001**
BMI	− 0.122	0.083	−	−
Wt	− 0.157	**0.025**	− 0.109	0.121
WC	− 0.160	**0.022**	0.107	0.128
SBP	− 0.091	0.195	− 0.028	0.691
DBP	− 0.179	**0.010**	− 0.134	0.057
TC^a^	− 0.136	0.053	− 0.130	0.064
TG^a^	− 0.151	**0.031**	− 0.114	0.106
HDL-C	0.003	0.971	0.064	0.364
FBG	− 0.224	**0.001**	− 0.204	**0.003**
TyG	− 0.186	**0.008**	− 0.149	**0.034**
VAI^a^	− 0.123	0.079	− 0.076	0.283
LAP^a^	− 0.171	**0.015**	− 0.116	0.100
MetS-IR^a^	− 0.117	0.096	− 0.012	0.864
TG/HDL-C^a^	− 0.117	0.097	− 0.069	0.331
PTH^a^	− 0.145	**0.040**	− 0.130	0.069

Adjusted 1 was analyzed after controlling for sex and age

Adjusted 2 was analyzed after controlling sex, age and BMI

Values were in bold when P < 0.05

^a^Skewed distributions including 25(OH)D were logarithmically transformed

### Odds ratio for MetS and its components in terms of the tertiles of serum 25(OH)D levels in all subjects

All subjects were stratified into trisections according to 25(OH)D tertiles (lowest: < 12.50 μg/mL; median: ≥ 12.50 to < 15.95 μg/mL; highest: ≥ 15.95 μg/mL). As shown in Table 3, the probability of the MetS in subjects in the lowest tertile of 25(OH)D levels was 1.179-fold higher than those in the highest tertile after adjusted age and sex (Model 1; OR = 2.179; 95% CI 0.982–4.833; *P* = 0.045). However, this phenomenon was abolished after further adjusted for BMI (Model 2). As MetS consists of multiple components, the relationship between 25(OH)D levels and each component of MetS were further proceeded by multiple logistic regression method. With respect to FBG, the participants in the lowest tertile of 25(OH)D levels had higher odds for presenting elevated FBG based on Model 1(OR = 2.954; 95% CI 1.408–6.200; *P* = 0.004), and this relationship still existed with further adjustment of BMI (Model 2; OR = 2.854; 95% CI 1.340–6.079; *P* = 0.007). However, 25(OH)D tertiles were not associated with the risk of the increased TG, the reduced HDL-C and the elevated BP.

**Table 3:** OR (95% CI) for MetS and its components in terms of 25(OH)D tertiles in all subjects

Measurements	25(OH)D tertiles		
	Lowest (n = 73) OR (95% CI)	Median (n = 68) OR(95% CI)	Highest (n = 70) OR(95% CI)
Range(μg/mL)	< 12.50	≥ 12.50 to < 15.95	≥ 15.95
MetS			
Model 1	2.179 (0.982, 4.833)	1.695 (0.767, 3.748)	1.00 (reference)
*P*	**0.045**	0.192	
Model 2	2.297 (0.611, 8.631)	1.919 (0.482, 7.639)	1.00 (reference)
*P*	0.218	0.355	
FBG (≥ 5.6 mmol/L)			
Model 1	2.954 (1.408, 6.200)	1.466 (0.707, 3.039)	1.00 (reference)
*P*	**0.004**	0.304	
Model 2	2.854 (1.340, 6.079)	1.432 (0.681, 3.012)	1.00 (reference)
*P*	**0.007**	0.344	
TG (≥ 1.70 mmol/L)			
Model 1	1.825 (0.886, 3.759)	1.423 (0.694, 2.916)	1.00 (reference)
*P*	0.103	0.335	
Model 2	1.672 (0.783, 3.580)	1.362 (0.636, 2.918)	1.00 (reference)
*P*	0.184	0.427	
HDL-C (< 1.03 mmol/L for males or < 1.29 mmol/L for females)			
Model 1	1.118 (0.567, 2.207)	0.866 (0.440, 1.706)	1.00 (reference)
*P*	0.748	0.678	
Model 2	0.870 (0.410, 1.844)	0.751 (0.358, 1.573)	1.00 (reference)
*P*	0.716	0.447	
BP (SBP ≥ 130 mmHg and/or DBP ≥ 85 mmHg)			
Model 1	1.891 (0.897, 3.984)	1.068 (0.518, 2.202)	1.00 (reference)
*P*	0.094	0.858	
Model 2	1.506 (0.612, 3.704)	0.923 (0.385, 2.211)	1.00 (reference)
*P*	0.373	0.858	

Model 1 was analyzed after controlling for **sex** and age

Model 2 was analyzed after controlling sex, age and BMI

Values were in bold when *P* < 0.05

### The levels of 25(OH)D were gradually decreased with the increase of MetS score in all subjects

**Fig. 1:**
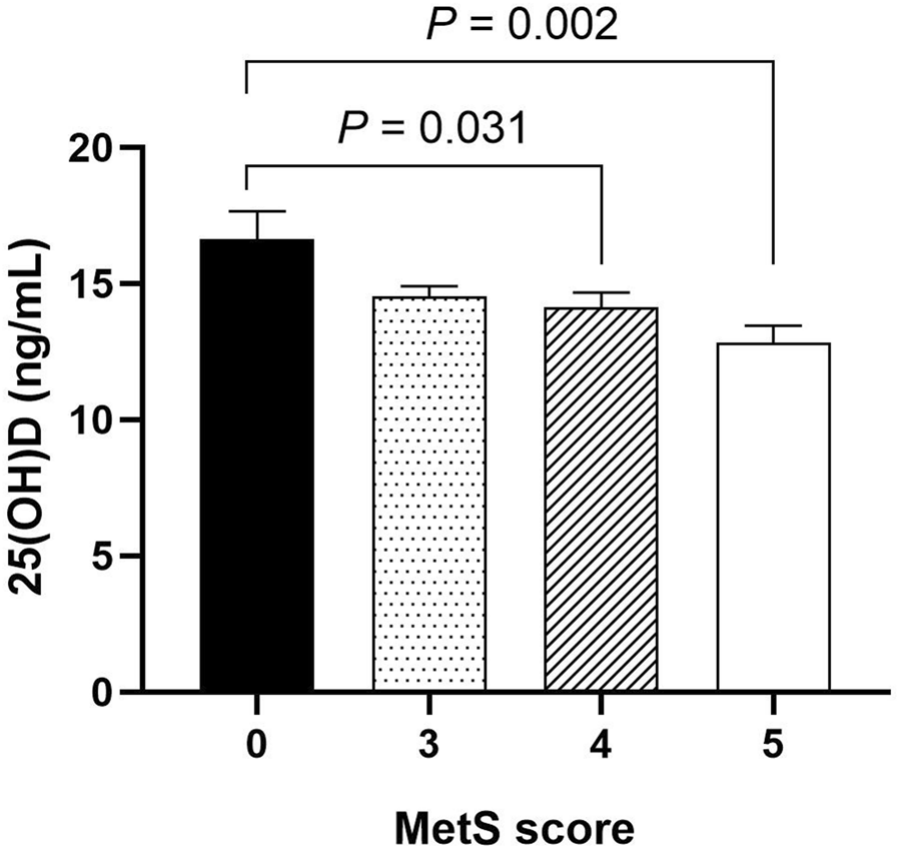
The levels of 25(OH)D were gradually decreased with the increase of MetS score in all subjects. Data were expressed as mean ± SD

The diagnosis of MetS requires the existence of central obesity plus at least two out of four metabolic components, and the severity of MetS increased as these components mounting. We next calculated the MetS scores, which was defined as the number of MetS components (range, 0–5) to reflect the metabolic status of MetS patients. As displayed in Figure 1, compared with 0 score group (controls), 25(OH)D levels in patients with 4 scores were significantly decreased (14.14 ± 0.54 *vs.* 16.63 ± 1.03 ng/mL, *P* = 0.031). As scores increased, 25(OH)D levels were further declined to 12.82 ± 0.64 ng/mL in patients with 5 scores (*P* = 0.002).

### Odds ratio for MetS scores in terms of the tertiles of serum 25(OH)D levels in MetS patients

Next, we investigated the relationship between 25(OH)D tertiles and MetS scores in the MetS patients utilizing ordinal logistic regression model. As shown in Table 4, lower 25(OH)D levels were correlated with higher odds for presenting more MetS components. Compared with the patients in the highest tertile of 25(OH)D levels (≥ 15.37 ng/mL), patients in the lowest tertlile of 25(OH)D levels (< 12.30 ng/mL) had 2.241-fold higher probability of gaining one MetS score after adjusting for sex and age (Model 1; OR: 3.241; 95% CI 1.428–7.340; *P* = 0.005). The association remain significant after further adjustment of BMI (Model 2; OR: 3.152; 95% CI 0.721–7.164; *P* = 0.006).

**Table 4:** Odds ratio (95% CI) for MetS scores according to 25(OH)D tertiles in MetS patients

Measurements	Tertiles		
	Lowest (n = 49) OR (95% CI)	Median (n = 51) OR (95% CI)	Highest (n = 51) OR (95% CI)
Range(μg/mL)	< 12.30	≥ 12.30 to < 15.37	≥ 15.37
MetS scores			
Model 1	3.241(1.428, 7.340)	1.527(0.705, 3.307)	1.00(reference)
*P*	**0.005**	0.283	
Model 2	3.152(0.721, 7.164)	1.458(0.671, 3.168)	1.00(reference)
*P*	**0.006**	0.341	

Model 1 was analyzed after controlling for age and sex

Model 2 was analyzed after controlling age, sex and BMI

Values were in bold when *P* < 0.05

### Comparison of metabolic parameters in MetS patients at baseline and 1-year follow-up after intervention

All 151 MetS patients enrolled were received interventions including diet and exercise counseling. As shown in Table 5, MetS score of the participants were significantly dropped at 1-year follow-up (3.68 ± 0.98 *vs.* 3.20 ± 0.73 ng/mL, *P* < 0.001), Anthropometric variables of MetS patients including Wt, BMI, WC as well as FBG were also significantly decreased (*P* < 0.05). As for metabolic indices, TyG and MetS-IR were declined prominently compared with that at baseline. Interestingly, the 25(OH)D levels were notably elevated at 1-year follow-up compared with at baseline (16.00 ± 4.09 *vs.* 14.13 ± 3.65 ng/mL, *P* < 0.001) in MetS patients without vitamin D supplementation. Besides, the levels of PTH were decreased with significance when compared to that of baseline (39.27 ± 16.25 *vs.* 44.99 ± 13.27 mmol/L, P < 0.001).

**Table 5:** Comparison of clinical and biochemistry parameters at baseline and 1-year follow-up in MetS patients

Variables	Pre-intervention (n = 151)	Post-intervention (n = 151)	*P* value
MetS score	3.68 ± 0.70	3.17 ± 0.98	** < 0.001**
Wt (kg)	77.90 ± 11.86	73.37 ± 12.30	** < 0.001**
BMI (kg/m^2^)	29.25 ± 3.24	27.77 ± 3.44	** < 0.001**
WC (cm)			
Male	99.07 ± 7.28	95.68 ± 8.01	** < 0.001**
Female	89.25 ± 7.05	87.86 ± 7.15	**0.048**
SBP (mmHg)	141.83 ± 16.05	138.60 ± 17.36	**0.017**
DBP (mmHg)	90.61 ± 9.08	89.89 ± 11.23	0.405
TC (mmol/L)	4.91 (4.30, 5.73)	4.93 (4.37, 5.65)	0.445
TG (mmol/L)	1.80 (1.31, 2.45)	1.66 (1.14, 2.28)	0.241
HDL-C (mmol/L)			
Male	1.04 (0.89, 1.15)	1.01 (0.89, 1.14)	0.636
Female	1.18 (1.03, 1.29)	1.15 (1.07, 1.35)	0.352
LDL-C (mmol/L)	3.05 ± 0.74	3.20 ± 0.73	**0.007**
FBG (mmol/L)	5.56 ± 0.55	5.27 ± 0.61	**0.008**
UA	271.25 ± 83.00	278.19 ± 74.62	0.068
VAI			
Male	2.56 (1.97, 3.76)	2.52 (1.78, 3.29)	0.315
Female	2.64 (1.90, 3.73)	2.05 (1.48, 3.35)	0.790
LAP			
Male	67.33 (49.59, 90.90)	55.68 (39.12, 82.32)	0.054
Female	52.92 (38.16, 65.92)	40.83 (29.23, 62.34)	0.361
TyG	9.00 ± 0.52	8.83 ± 0.58	** < 0.001**
MetS-IR	45.59 (41.16, 50.23)	42.69 (38.94, 48.20)	** < 0.001**
TG/HDL-C	1.72 (1.12, 2.27)	1.50 (0.91, 2.16)	0.324
25(OH)D (ng/mL)	14.13 ± 3.65	16.00 ± 4.09	** < 0.001**
PTH (mmol/L)	43.1 (36.68. 52.63)	37.35 (27.60, 50.17)	** < 0.001**

Data were expressed as ratio for categorical variables and mean ± SD for normally distributed continuous variables, median (IQR) for skewed distributed continuous variables. *P* values are from Paired T-test. Values in bold are of significance at P < 0.05

### Associations between the changes of 25(OH)D levels and metabolic parameters in MetS patients after 1-year intervention

Next, the longitudinal associations between the changes of 25(OH)D and metabolic parameters were investigated in MetS patients using liner mixed effect model. As shown in Table 6, after adjusting for sex, age and BMI, the increase of 25(OH)D levels were significantly related to the decrease of MetS score (β: − 1.512; 95% CI − 2.585 to − 0.440; *P* = 0.008) and lipid related traits including TC (β: − 0.271; 95% CI − 0.473 to − 0.069; *P* = 0.009) and LDL-C (β: − 1.832; 95% CI − 2.945 to − 0.718; *P* = 0.001). Besides, it was also associated with elevated HDL-C levels (β: 0.168; 95% CI 0.192–0.317; *P* = 0.028), indicating that increased 25(OH)D levels were associated with the improved lipid profile. What’s more, the increase of 25(OH)D levels were notably contributed to the decrease of FBG (β: − 1.128; 95% CI − 1.967 to − 0.286; *P* = 0.009) and TyG (β: − 0.700; 95% CI − 1.506 to − 0.107; *P* = 0.048). Taken together, these results suggested that increased levels of 25(OH)D may play a role in the overall amelioration of lipid and glucose metabolism.

**Table 6:** Associations between the changes of 25(OH)D levels and metabolic parameters in MetS patients with 1-year intervention by using liner mixed effect model

Variables	Adjusted	
	β (95% CI)	*P*
MetS score	− 1.512 (− 2.585, − 0.440)	**0.008**
WC	− 6.048 (− 14.175, 2.078)	0.123
SBP	− 4.599 (− 28.415,19.218)	0.703
DBP	− 8.617 (− 22.034, 4.800)	0.299
TC^a^	− 0.271(− 0.473, − 0.069)	**0.009**
TG^a^	− 0.210 (− 0.563, 0.144)	0.242
HDL-C	0.168 (0.192, 0.317)	**0.028**
LDL-C	− 1.832 (− 2.945, − 0.718)	**0.001**
FBG	− 1.128 (− 1.967, − 0.286)	**0.009**
TyG	− 0.700 (− 1.506, 0.107)	**0.048**
VAI^a^	− 0.071(− 0.512,0.371)	0.866
LAP^a^	− 0.287 (− 0.667,0.921)	0.188
MetS-IR^a^	0.045 (− 0.059,0.148)	0.397
TG/HDL-C^a^	− 0.043 (− 0.479,0.393)	0.846
PTH^a^	− 0.079 (− 0.282,0.123)	0.440

Models were adjusted for age, sex and BMI

^a^Skewed distributions including 25-hydroxy25(OH)D were logarithmically transformed. Values in bold are of significance at P < 0.05

## Discussion

In our present study, we found that serum 25(OH)D levels were significantly decreased in MetS patients, and inversely associated with MetS and its components both cross-sectionally and longitudinally in northern Chinese middle-aged subjects without vitamin D supplementation. Participants with the lowest tertlile of 25(OH)D levels were much more likely to present MetS and hyperglycemia in all subjects, and one extra MetS score gain in MetS patients than those in the highest tertile of 25(OH)D levels. After 1 year of diet and exercise counselling, metabolic aberrations of MetS patients were greatly ameliorated, and 25(OH)D levels were notably elevated. Further analysis using linear mixed effect model, which combined data from MetS patients before and after intervention, demonstrated that the increased 25(OH)D levels were negatively associated with adiposity traits, lipid profile and glucose metabolism.

25(OH)D is a well-known steroid hormone, which is essential for the maintenance of calcium homeostasis and for bone health [27]. However, due to the lack of outdoor activities and unhealthy eating habits, vitamin D deficiency has now become a global health burden regardless of regions, races, age and sex [4, 28]. As risk factors are highly overlapped, many studies have revealed that lower 25(OH)D status were more commonly identified in individuals with MetS. In our cross-sectional findings, we found that serum 25(OH)D levels in MetS patients were significantly lower than controls (the vitamin D deficiency rate among MetS patients was 92.05%). In consistence with our results, a study conducted in elderly Chinese individuals (aged 65–112 years) from 8 major cities also found that the serum 25(OH)D levels of the participants with MetS were significantly lower than that of the individuals without MetS [29]. Similar results were also obtained in Chinese children and young adolescents in Beijing [5]. Moreover, in our longitudinal analyses, it was observed that after 1-year, the overall metabolic status of MetS patients were attenuated, and 25(OH)D levels were remarkably elevated, suggesting that the improvement of MetS may closely associated with increased 25(OH)D levels.

In the present study, it was shown that participants with lower vitamin D had higher odds of having MetS after adjusting for age and sex. Consistent with our results, a study enrolled 380 Malay adults found that 25(OH)D insufficiency was independently associated with an increased risk of having metabolic syndrome [30]. Calculated by counting the number of MetS components, MetS score have been utilized to assess the severity of MetS [25]. Studies have shown that MetS scores perform better in predicting the risk for comorbidities, including CVD and diabetes than the sole diagnosis of MetS [31–33]. To our knowledge, this is the first study to reveal the relationship between MetS score and 25(OH)D levels particularly in northern Chinese middle-aged population. In this study, we found that 25(OH)D levels was significantly negatively correlated with MetS score in all subjects, and 25(OH)D levels were gradually decreased with MetS score mounting. Interestingly, we further observed that lower 25(OH)D levels in MetS patients were notably associated with the probability for exhibiting higher MetS score even after adjustments for age, sex and BMI. Our finding was supported by researches conducted in Korean populations, which demonstrated that serum 25(OH)D levels were decreased significantly with an increase in MetS score [34, 35]. Longitudinally, in our present study, MetS score were remarkably decreased after 1 year of diet and exercise intervention. Further analysis by using linear mixed effect model showed that the increases of 25(OH)D levels were inversely associated MetS score in MetS patients. In consistence with our findings, a study conducted in Canada revealed that after 1-year of lifestyle counselling (without compulsive vitamin D supplementation), the serum 25(OH)D concentrations of the participants were also elevated, and statistically significant inverse relationships of the increases in 25(OH)D with risk for MetS were observed [36]. Collectively, these results indicated that the increase of 25(OH)D levels in MetS patients may be conversely beneficial for the improvement of metabolic dysfunctions in addition to the diet and exercise intervention.

Obesity is one of the main characteristics of MetS. Several studies have reported the obese individuals were more likely to present low 25(OH)D levels. Pereira-Santos M, et, al found that vitamin D deficiency was highly prevalent in obese subjects [37], and another meta-analysis combined 36 cross-sectional studies with 257, 699 participants revealed that vitamin D status was inversely related to the incidence of abdominal obesity [38]. In accordance with these studies, our data showed that MetS patients had higher Wt, WC, BMI and lower 25(OH)D levels. It has been reported that vitamin D is highly fat-soluble, hereby the comparatively low levels of 25(OH)D of MetS patients were derived from the vitamin sequestration in excess adipose tissue, and volumetric dilution in the large fat mass [39]. After 1-year intervention, the BMI and WC of MetS patients was significantly decreased and the circulating 25(OH)D levels was notably elevated. One possible explanation is that the stored 25(OH)D in adipose tissue, especially in visceral fat, was being released following the fat mass reduction. In agreement with our result, Buscemi S, et al. also found that significant decrease in BMI, WC and fat mass (FM) induced by a very low-calorie ketogenic diet resulted in the increased serum 25(OH)D levels [40]. A meta-analysis which recruited 18 clinical trials also demonstrated that serum vitamin D levels were increased with weight and FM loss [41], further supporting the hypothesis that increases in serum 25(OH)D levels would be a result from direct mobilization of stores into the circulation upon decrease of FM in the obese subjects. Besides, in the present study, the follow-up diet and exercise counselling including healthy diet and outdoor activities also contribute to the increase of serum 25(OH)D levels.

Next, our correlation analysis revealed that 25(OH)D levels were significantly negatively related to TG after adjusted for sex and age, although the relations were attenuated after further adjustment of BMI. Similarly, there are studies that failed to establish solid association between 25(OH)D levels and lipid metabolism-related traits after adjustments for BMI in overweight or obese children and adolescents [5, 42]. However, other studies found that in middle-aged or elderly population, where the overall BMI were within normal range, the inverse relationship remained substantial after adjusting for BMI [43, 44]. Taken together, these results suggested that the cross-sectional inverse relationships between 25(OH)D and lipid profile may be dependent on BMI, especially in overweight or obese population. Interestingly, in our longitudinal study with 1-year follow-up, MetS patients exhibited significant improvement in 25(OH)D levels. Longitudinal correlations showed that the increase of serum 25(OH)D levels was negatively related to TC and LDL-C, and positively related to HDL-C after adjusted for age, sex and BMI, suggesting this longitudinal relationship was independent of BMI. However, several interventional studies have elaborated that vitamin D supplementation in humans showed controversial effects in lipid metabolism [45]. Therefore, whether vitamin D is a casual factor for the improvement of dyslipidemia in MetS, and how BMI interplays during the process still need to be further elucidated. In addition, LDL-C levels of MetS patients were found to be increased from 3.05 ± 0.74 to 3.20 ± 0.73 mmol/L after 1-year intervention. However, both of these values were within normal range in accordance with the Chinese Guidelines on Prevention and Treatment of Dyslipidemia (2016 Edition) and the Adult Treatment Panel III [46]. Therefore, the clinical significance of LDL-C elevation was limited.

With regard to the associations between serum 25(OH)D levels and glucose status in MetS patients, our data revealed that 25(OH)D levels were strongly negatively associated with FBG. Besides, subjects in lowest tertile of 25(OH)D levels were 2.8 times more likely to exhibit the elevated FBG after adjusted for age, sex and BMI. In accordance with our results, a study conducted in Korean adolescents revealed that vitamin D deficiency was associated with a 2.07-fold higher risk of the elevated FBG in individuals with MetS [47]. However, there were studies which did not establish such significant associations between the two factors [48, 49]. Even in studies which implemented over 8 weeks of vitamin D supplementation did not show significant improvement in FBG in subjects with prediabetes, obesity or hypertension [50, 51]. Beside hyperglycemia, IR represents a major pathological cause of diabetes. In recent years, metabolic indices including TyG, VAI, LAP, MetS-IR and TG/HDL-C have been used for evaluation of IR in diverse populations [13, 16, 52]. In our study, we found that compared with the controls, these indices were significantly elevated in MetS patients, and the TyG and LAP were remarkably inversely related to 25(OH)D levels independent of age, sex and BMI. One study reported in 2014 that in patients with hyperglycemia, 25(OH)D levels were negatively associated with LAP, and subjects with higher LAP exhibit elevated risk for vitamin D deficiency [53]. After 1-year, we found that TyG and MetS-IR of MetS patients showed notable improvements, and the increases of 25(OH)D levels notably and independently contributed to the decrease of TyG.

PTH has been shown to modulate calcium and energy homeostasis in an interactive way with vitamin D. It was found in our present study that PTH levels were negatively related to 25(OH)D levels at baseline, though the significance of which was attenuated after further adjustment of BMI. In accordance with our results, studies have also revealed a strong negative association between PTH and 25(OH)D levels [54], and elevated PTH were associated with increased risk of MetS or its components [55, 56]. It is reported that PTH possesses the ability of suppressing the activity of lipoprotein lipase in adipocytes [57] and it is thus reasonable to suggest that vitamin D may alleviate MetS by decreasing PTH levels. Moreover, longitudinally, PTH levels were significantly decreased at 1-year follow-up, but the decrease of which was not significantly associated with the increase of 25(OH)D levels.

The longitudinal design, the homogeneity of samples and multiply utilized metabolic parameters and indices are the strengths of this study. However, some limitations still need to be illustrated. Several other factors, such as sun exposure and dietary intake, which may influence serum 25(OH)D levels were not assessed in this study, although the study samples were collected in the same season of the year and the lifestyle of each individual were unlikely to change profoundly within 1 year. Moreover, another limitation of this study is the absence of a simultaneously recruited MetS group without interventions. We suggested our study warrants further perspective cohort studies for confirmation.

## Conclusions

In conclusion, cross-sectionally, our study revealed that 25(OH)D levels were generally decreased in patients with MetS, and it was inversely related to MetS risk profile. Longitudinally, after 1-year, the notable improvements of metabolic status and the elevated 25(OH)D levels in MetS patients were observed, and the increases of 25(OH)D levels were positively related to the amelioration of MetS.

## Data Availability

All data generated are included in this published article.

## Abbreviations

MetS: Metabolic syndrome
BMI: Body mass index
Wt: Weight
Ht: Height
WC: Waist circumference
SBP: Systolic blood pressure
DBP: Diastolic blood pressure
TC: Total cholesterol
TG: Triglycerides
HDL-C: High-density lipoprotein-cholesterol
FBG: Fasting blood glucose
UA: Uric acid
Cr: Creatinine
BUN: Blood urea nitrogen
VAI: Visceral adiposity index
LAP: Lipid accumulation product
TyG: Product of triglycerides and glucose
MetS-IR: Metabolic score for insulin resistance
25(OH)D: 25-Hydroxyvitamin D
PTH: Parathyroid hormone
OR: Odds ratio
